# HiGATE: hierarchical graph attention for multi-scale tissue encoder in computational pathology

**DOI:** 10.3389/fonc.2026.1798040

**Published:** 2026-05-25

**Authors:** Imam Dad, Jianfeng He, Tao Shen

**Affiliations:** 1 Faculty of Information Engineering and Automation, Kunming University of Science and Technology, Kunming, Yunnan, China; 2 Department of Colorectal Surgery, The Third Affiliated Hospital of Kunming Medical University, Kunming, Yunnan, China

**Keywords:** computational pathology, cross-level attention, Explainable AI, graph attention networks, hierarchical graph neural networks, histopathological analysis, multiscale learning

## Abstract

**Background:**

Histopathological diagnosis is inherently a multi-scale reasoning process, where pathologists seamlessly integrate cellular morphology with tissue architecture. Yet, computational models remain fragmented by analyzing cells and tissues in isolation, missing the diagnostic synergy that emerges from their interplay. This disconnect limits both predictive accuracy and clinical trust.

**Methods:**

We introduce HiGATE (Hierarchical Graph Attention Tissue Encoder), a biologically-inspired framework that unifies cellular and tissue-level analysis through a novel dual-graph architecture. Unlike prior hierarchical models that rely on static, unidirectional information flow, HiGATE introduces a bidirectional Cross-Level Attention mechanism enabling dynamic, context-aware communication where cellular details inform tissue organization and architectural context refines cellular representations. The framework incorporates learnable, spatially-constrained graph construction via differentiable pooling with spatial regularization, adaptively capturing tissue heterogeneity rather than relying on fixed heuristics. Multi-modal nuclear features integrate domain-adapted visual semantics (DINOv2), morphological shape descriptors, and fine-grained morphometrics (StarDist). We validate HiGATE across four diverse datasets spanning multiple tasks: PanNuke (nuclei classification with dual hierarchical levels: 5 nuclear types and 19 tissue types), MoNuSeg (segmentation feature transfer), DigestPath (colon polyp classification), and TCGA-BRCA (whole-slide breast cancer grading).

**Results:**

On the PanNuke benchmark, HiGATE achieves state-of-the-art performance with 91.3% accuracy and an F1-score of 0.896 for nuclei classification, while also achieving 85.4% accuracy for tissue-type classification across 19 cancer types. The framework demonstrates exceptional cross-dataset generalization: MoNuSeg segmentation (Dice = 0.841), DigestPath classification (accuracy = 0.872), and TCGA-BRCA WSI-level grading (accuracy = 0.854). Similarly, at a clinically relevant high-sensitivity operating point (recall = 0.95), HiGATE maintains precision of 0.87—a 10.1% reduction in false positives over HACT-Net. A multi-reader study with five board-certified pathologists confirms the clinical relevance of HiGATE’s integrated multi-scale explanations (mean diagnostic relevance = 4.1/5.0, *p <* 0.001).

**Conclusion:**

HiGATE bridges the gap between high-performance AI and clinically actionable diagnostics by unifying predictive accuracy with transparent, pathologist-aligned reasoning. The bidirectional cross-scale attention mechanism constitutes a general contribution to hierarchical graph representation learning, with potential applications extending beyond computational pathology to any domain requiring sophisticated multi-scale relational reasoning. Our comprehensive validation across tasks and tissue types establishes HiGATE as a robust foundation for trustworthy diagnostic AI in personalized medicine.

## 1 Introduction

Histopathological image analysis remains the gold standard for cancer diagnosis, prognosis, and therapeutic decision-making (1). Whole Slide Images (WSIs), with their gigapixel resolution and complex tissue architectures, encode a wealth of diagnostic information across multiple biological scales; from subcellular details to tissue-level organization. However, manual analysis of WSIs is limited by diagnostic subjectivity, inter-observer variability, and pathologist cognitive overload (2). These challenges have been a key driver for the advancement of computational pathology, which leverages Artificial Intelligence (AI) to augment pathological workflows, improve diagnostic consistency, and enable precision oncology.

The trajectory of AI in histopathology has progressed through distinct methodological paradigms (3). Early approaches relied on handcrafted morphological features combined with traditional classifiers like Support Vector Machines (SVMs) (4, 5). However, these approaches struggled to capture the nuanced and heterogeneous patterns characteristic of cancer pathology. The advent of Deep Learning (DL), particularly Convolutional Neural Networks (CNNs), marked a transformative shift by enabling end-to-end feature learning directly from image pixels (6). CNNs have demonstrated success in tasks such as nuclei segmentation (7) and patch-level classification (8). However, their fundamental architectural limitation and local receptive fields restrict their ability to model long-range spatial dependencies and global tissue architecture, important for comprehensive diagnostic reasoning (9).

To overcome the context-blindness of CNNs, two complementary research directions have emerged. First, Vision Transformers (ViTs) address long-range dependency modeling through self-attention mechanisms applied to sequences of image patches (10, 11). Recent advances such as Swin Transformers (12) and TransPath (13) have demonstrated impressive performance by incorporating hierarchical attention windows and histology-specific pretraining. Hybrid architectures like Co-Scale Conv-Attentional Transformers (CoaT) (14) combine convolutional layers with attention mechanisms to integrate local inductive biases and global context. However, as noted in recent studies (15, 16), transformer-based methods treat image patches as sequence tokens without explicitly modeling the biological hierarchy of tissue or capturing spatial and topological relationships essential for diagnostic reasoning.

Second, Graph Neural Networks (GNNs) (17) offer a biologically intuitive framework by representing tissue as graphs where nodes correspond to biological entities (cells, regions) and edges encode spatial or functional relationships. GNN-based approaches have shown promise in capturing relational micro-structures for tasks such as cancer sub-class classification (18) and nuclei segmentation (19). However, a persistent limitation of most GNN methods is their confinement to a single scale, focusing either on cellular interactions or tissue-level patterns (7). This single-scale focus produces biologically incomplete representations that fail to model the intrinsic hierarchical organization of tissue, where diagnostic meaning emerges from the interplay between cytological details and architectural context.

This recognition has instigated the development of Hierarchical Graph Neural Networks (HGNNs) (20, 21), aimed to jointly model multiple biological scales. Recent advancements have seen innovative architectures such as Hierarchical Cell-toTissue Graph Neural Network (HACT-Net) (21) for multi-level entity graphs and Heterogeneous Graph Attention Networks (HAN) (22) bringing attention mechanisms across predefined scales. However, these hierarchical models exhibit fundamental limitations: (1) they rely on static or sequential bottomup processing with minimal top-down feedback, lacking the bidirectional integration that constantly shifts focus between cellular details and tissue architecture; (2) they depend on fixed graph construction heuristics (e.g., k-NN with predetermined thresholds, watershed segmentation) that are unable to adapt inherent tissue heterogeneity; and (3) they lack mechanisms for learnable, context-aware region formation, instead relying on predefined spatial partitions that may not align with biologically meaningful tissue compartments (23, 24).

Beyond predictive performance, the successful integration of AI into clinical practice hinges on interpretability and trust. In this regard, Explainable AI (XAI) has become indispensable in computational pathology, addressing the “black box” problem that encumbers clinical adoption (25, 26). For successful integration into clinical practice, AI models must offer interpretability that aligns with the pathologist’s diagnostic reasoning. Current XAI techniques, such as gradient-based attribution methods [e.g., Gradient-weighted Class Activation Mapping (Grad-CAM) (27, 28)] or graph explainers [e.g., GNNExplainer (29)], typically operate at a single scale, producing explanations that are either narrowly focused on isolated cells or overly broad tissue-level saliency maps. This fragmentation creates a critical gap between computational output and clinically actionable insight.

Extensive evaluation on the PanNuke dataset (30) shows that HiGATE achieves state-of-the-art performance, significantly outperforming recent hierarchical models such as HACT-Net (31) and HAN (22), as well as transformer-based approaches. The framework also demonstrates strong generalizability in cross-dataset validation on MoNuSeg (32), DigestPath (33), and TCGA-BRCA (34). By unifying dynamic cross-hierarchical reasoning with high accuracy and interpretability, HiGATE advances toward clinically actionable, trustworthy diagnostic AI while contributing a novel architectural module to the broader field of hierarchical graph learning.

## 2 Related work

Computational pathology has evolved to emulate the pathologist’s diagnostic integration of nuclear and architectural features. The field has progressed from handcrafted features to DL (6, 35), ViTs (36), and GNNs (37), with current efforts focusing on hierarchical, multi-scale modeling (38). Recent comprehensive studies (39, 40) highlight the need for interpretable, multi-scale frameworks in computational pathology. Additionally, emerging approaches such as hypergraph neural networks (41) and contrastive learning frameworks (42) demonstrate the growing importance of integrating morphological and molecular information for predictive modeling. To address these critical problems, HiGATE incorporates an integrated multi-scale design with bidirectional attention.

### 2.1 From handcrafted features to DL: the first revolution

The initial computational forays into histopathology were grounded in handcrafted feature engineering. Early works extracted quantitative descriptors of nuclear morphology (e.g., area, eccentricity) (43), chromatin texture (44), and architectural patterns (e.g., glandular formation) (44) to train traditional classifiers like SVMs (4) and Random Forests (RF) (45). While providing interpretable features, these methods proved inadequate for the vast complexity and heterogeneity of WSIs, struggling to capture the nuanced, high-dimensional patterns indicative of disease (46, 47).

DL, and in particular CNNs, has revolutionized disciplines such as econometrics, biology, and finance by enabling the extraction of hierarchical representations from complex datasets (48). In computational pathology, CNNs have dramatically improved performance on core diagnostic tasks including tumor detection (49), cancer grading (50), and histological subtyping (51) by learning feature hierarchies directly from pixel data. This progress was accelerated by the widespread adoption of Transfer Learning (TL), where architectures such as ResNet (52) and EfficientNet (53), pretrained on large natural image datasets, became standard practice (54). Subsequent works, including attention-based pooling mechanisms such as Clustering-constrained Attention Multiple Instance Learning (CLAM) (55), further advanced the field by enabling weakly supervised slide-level classification through intelligent aggregation across hundreds of thousands of image patches. Despite these advances, CNNs are fundamentally limited by their architectural inductive bias. Built upon fixed convolutional kernels, they excel at local pattern recognition such as identifying mitotic figures (56) but struggle to model long-range spatial dependencies and the global tissue architecture that underpins pathological diagnosis (57). This core constraint persists even after the introduction of architectural refinements like dilated convolutions (58) and multiscale pyramidal networks (59), which partially mitigate the inability to capture holistic tissue organization.

### 2.2 Vision Transformers: capturing global context

ViTs (36) emerged as an alternative transformation, treating images as sequences of patches and applying self-attention to model global context. This is advantageous for histopathology, where diagnostic patterns often emerge from interactions across large tissue regions (36). Subsequent adaptations, including SWIN Transformers (12) with hierarchical attention windows and TransPath (13) pretrained on histology data, have shown high performance in tasks requiring contextual integration, such as metastasis detection (60) and head-to-head comparison between two DL architectures CNNs and ViTs in the specific domain of cancer imaging (61).

Recent advances have adapted ViTs to address specific challenges in histopathology. Hierarchical Vision Transformers (HVT) (62) process multi-resolution feature maps, while token merging techniques (63) alleviate the computational burden of gigapixel WSIs. Hybrid architectures, such as Co-Scale Conv-Attentional Transformers (CoaT) (14), combine convolutional layers with attention mechanisms to integrate local inductive biases and global context. Despite these innovations, ViTs treat image patches as sequence tokens without explicitly modeling the biological hierarchy of tissue such as cells, glands, and region or capturing their spatial and topological relationships, which are essential for diagnostic reasoning (15, 16, 60).

### 2.3 GNNs: a biologically-inspired paradigm

GNNs offer a more intuitive abstraction for tissue analysis by modeling biological entities as nodes and their relationships as edges in a graph *G* = (*V, E*) (64, 65). This framework accommodates the irregular, relational structure of tissue, enabling sophisticated analysis of spatial interactions and microenvironmental cues (7).

#### 2.3.1 Cellular-graph approaches

A dominant paradigm in computational pathology constructs cell graphs from nuclear segmentation masks (66) where each node represents an individual cell annotation with morphological, textural, and stain-based features (67). Early GCNs have shown high accuracy in breast cancer grading by modeling pairwise cell-cell interactions (68). Recent methods have advanced this approach through more sophisticated message passing architectures. For example, edge-conditioned convolutions are modeled to diverse types of cellular interactions (69), while the introduction of CytoCommunity (70) directly maps cellular phenotypes to tissue-level spatial organization via hierarchical graph pooling that bypasses intermediate clustering steps. CytoCommunity has successfully identified high-risk tumor-associated cellular neighborhoods and revealed rewired spatial communication networks.

Despite these advancements, two principal limitations constrain current cellular graph approaches. First, their operation at a single, cellular scale precludes the explicit integration of higher-order architectural patterns that are critical for pathological assessment. Second, their reliance on static graph construction heuristics [e.g., fixed radius neighborhood definitions (71)] lacks the adaptability necessary to model the heterogeneous cellular densities and organizational patterns observed across varying tissue types and disease states.

#### 2.3.2 Tissue-graph and hierarchical approaches

To capture higher-order tissue organization, tissue-graph models interactions between larger architectural units rather than individual cells. In these approaches, graph nodes may represent super pixels (72), glandular structures (73), or semantically segmented tissue patches (74), with edges encoding spatial adjacency, functional correlation, or morphological similarity. Prominent models such as Smoothed GraphEnhanced Multi-Instance Contrastive Learning (SG-MuRCL) (75) apply GNNs to patch-level graphs to enable slide-level classification through multi-instance contrastive learning.

The logical extension of tissue-graph modeling is hierarchical representation, which aims to bridge information across biological scales. Hierarchical models, such as HACT-Net, employed a two-level graph (cellular and regional) but relied on static, rule-based region grouping (watershed segmentation). More recent architectures, including dynamic hierarchical attention pooling mechanisms (76), attempt to learn multi-scale representations adaptively.

##### Analysis of existing hierarchical models:

- HACT-Net (31): Employs unidirectional bottom-up aggregation from cells to tissue regions using static watershed segmentation. Lacks top-down feedback, preventing contextual refinement of cellular representations. Region formation is non-learnable and may not align with biologically meaningful compartments.
- HAN (22): Uses heterogeneous graph attention across predefined node types but relies on fixed graph construction and does not incorporate learnable hierarchical pooling.
- Dynamic Hierarchical Pooling (77): Introduces learnable pooling but lacks spatial regularization for anatomical coherence and bidirectional cross-scale attention.
- CytoCommunity (70): Maps cellular phenotypes to tissue organization but operates in an unsupervised manner without task-specific optimization for classification.

HiGATE addresses these limitations through: (1) symmetric bidirectional Cross-Level Attention enabling dynamic top-down and bottom-up information flow; (2) learnable differentiable pooling with spatial regularization for adaptive, anatomically coherent region formation; and (3) end-to-end optimization for classification with integrated multi-scale explainability.

### 2.4 Hypergraph and contrastive learning approaches for spatial transcriptomics

Beyond HGNNs, recent advances have explored hypergraph architectures and contrastive learning strategies for integrating histology images with molecular data.

One notable approach is HyperGraph Gene Expression Prediction (HGGEP), a hypergraph neural network for predicting gene expression from histology images (41). HGGEP uses a gradient enhancement module to capture cell morphological information. It extracts multi-stage features from a lightweight backbone. A hypergraph association module then models higher-order relationships across features at different stages. This enables joint representation of complex spatial patterns. The hypergraph approach shares conceptual similarities with HiGATE’s hierarchical graph construction. Both aim to capture multi-scale relationships: HGGEP through hyper edges connecting multiple features, and HiGATE through symmetric bidirectional attention across cellular and tissue graphs.

Another recent framework is Histology-Enhanced Contrastive Learning for Imputation of Profiles (HECLIP) (42). It uses a contrastive learning strategy with an image-centric contrastive loss. HECLIP aligns histology image patches with transcriptomic profiles. It prioritizes image encoder optimization for accurate gene expression imputation from histology alone. The framework demonstrates strong performance in retrieving biologically relevant gene expression patterns. While HECLIP focuses on cross-modal alignment rather than explicit multi-scale tissue modeling, its image-centric representation learning complements HiGATE’s approach to feature extraction.

Collectively, these works underscore a growing trend in computational pathology toward modeling complex, multi-scale relationships, whether through hypergraphs (HGGEP), cross-modal alignment (HECLIP), or hierarchical attention (HiGATE). They highlight the importance of integrating morphological and molecular information for advancing diagnostic and predictive modeling.

### 2.5 The imperative for adaptive, multi-scale integration

Histopathological diagnosis necessitates reasoning across multiple biological scales, where individual cellular features such as nuclear pleomorphism (78) derive diagnostic significance from their architectural context. However, current multi-scale approaches remain fundamentally limited: while multi-stream networks process different image magnifications in parallel (79) and late-fusion methods combine features from separate scales (80), both lack explicit mechanisms for cross-scale feature interaction and spatial grounding. Attention-based frameworks, such as MS-CLAM (81), enable cross resolution patch interactions but operate at a global feature level without preserving explicit spatial-graphical relationships between biological entities. Truly integrative multi-scale graph learning remains underdeveloped, with existing methods relying on fixed, predefined hierarchies that cannot adapt to tissue heterogeneity (82) or employing static topological designs that limit granularity and flexibility (83). Collectively, these limitations underscore the need for adaptive, efficient, and interpretable multi-scale graph frameworks in computational pathology.

### 2.6 Explainable AI: bridging the trust gap in clinical translation

The intrinsic opacity of complex AI models constitutes a formidable impediment to their clinical adoption in pathology, where interpretability is paramount for diagnostic trust (84). Explainable AI (XAI) has emerged as a critical discipline to ameliorate this trust deficit by rendering model decisions transparent and interpretable (85). *Post-hoc* attribution methods, including Grad-CAM (27) and its histopathology-specific adaptations (86, 87), generate saliency maps that ostensibly highlight influential image regions. Nevertheless, these approaches yield single-scale, visually diffuse explanations that fail to elucidate the underlying relational logic of a diagnosis—for instance, connecting specific atypical cellular formations to the disordered glandular architecture they collectively constitute.

Concept-based paradigms, such as Concept Bottleneck Models (88), map predictions to human-interpretable clinical concepts like nuclear pleomorphism (89), yet they necessitate extensive expert annotations that are often prohibitively costly to procure (90). Within graph-based learning, explainers such as GraphXAIN (91) and GraphXIA (92) identify salient subgraphs or nodes, but their *post-hoc* application and confinement to single-scale explanations fragment the multi-scale rationale essential for comprehensive pathological reasoning. An emerging paradigm of inherently interpretable or self-explaining architectures embeds transparency directly into model design (93); in graph learning, this may manifest through attention weights signifying node or edge importance (94). However, contemporary self-explaining graph neural networks do not natively generate explanations that traverse multiple hierarchical levels. HiGATE addresses this critical lacuna through an integrated explainability framework that synergistically combines gradient attribution, perturbation analysis, and conservation checked Layer-wise Relevance Propagation (LRP), thereby establishing traceable connections between cellular-level evidence and tissue-level diagnostic conclusions.

### 2.7 Research gaps and contributions

Based on our literature analysis, we identify four key challenges in computational pathology:

- Scale Integration Gap: Models analyze cells and tissues separately, missing their diagnostic synergy. Existing hierarchical approaches (e.g., HACT-Net) use unidirectional bottom-up aggregation without top-down refinement.
- Representation Rigidity: Graph methods rely on static heuristics (fixed thresholds, grids, watershed) that cannot adapt to tissue heterogeneity. Learnable region formation remains unexplored.
- Explanation Disconnect: Current XAI methods produce single-scale rationales, failing to link cellular features to their architectural context.
- Benchmarking Limitation: Most models are evaluated on single datasets, lacking cross-task validation (classification, segmentation, WSI analysis). To address these, we introduce HiGATE with five key contributions:
  - Bidirectional Cross-Level Attention: Unlike HACT-Net’s unidirectional flow, our symmetric attention enables dynamic information exchange both bottom-up (cells → tissue) and top-down (tissue → cells), allowing mutual refinement across scales.
  - Learnable Spatially-Constrained Graph Construction: We replace fixed heuristics (k-NN, watershed) with differentiable graph pooling and spatial regularization, enabling adaptive, biologically-meaningful region formation.
  - Comprehensive Cross-Dataset Validation: We evaluate across four datasets and three tasks: PanNuke (classification with dual hierarchy), MoNuSeg (segmentation), DigestPath (colon classification), and TCGA-BRCA (WSI grading), demonstrating broad generalizability.
  - Rigorous Baseline Comparison: All graph-based (HACT-Net, HAN, HGT) and transformer-based (Swin, TransPath) baselines are reimplemented with identical features and training protocols for fair comparison.
  - Enhanced Explainability Validation: A multi-reader study with five pathologists on 150 patches, with statistical testing, validates the clinical relevance of our multi-scale explanations.

## 3 Methodology

**Figure 1:**
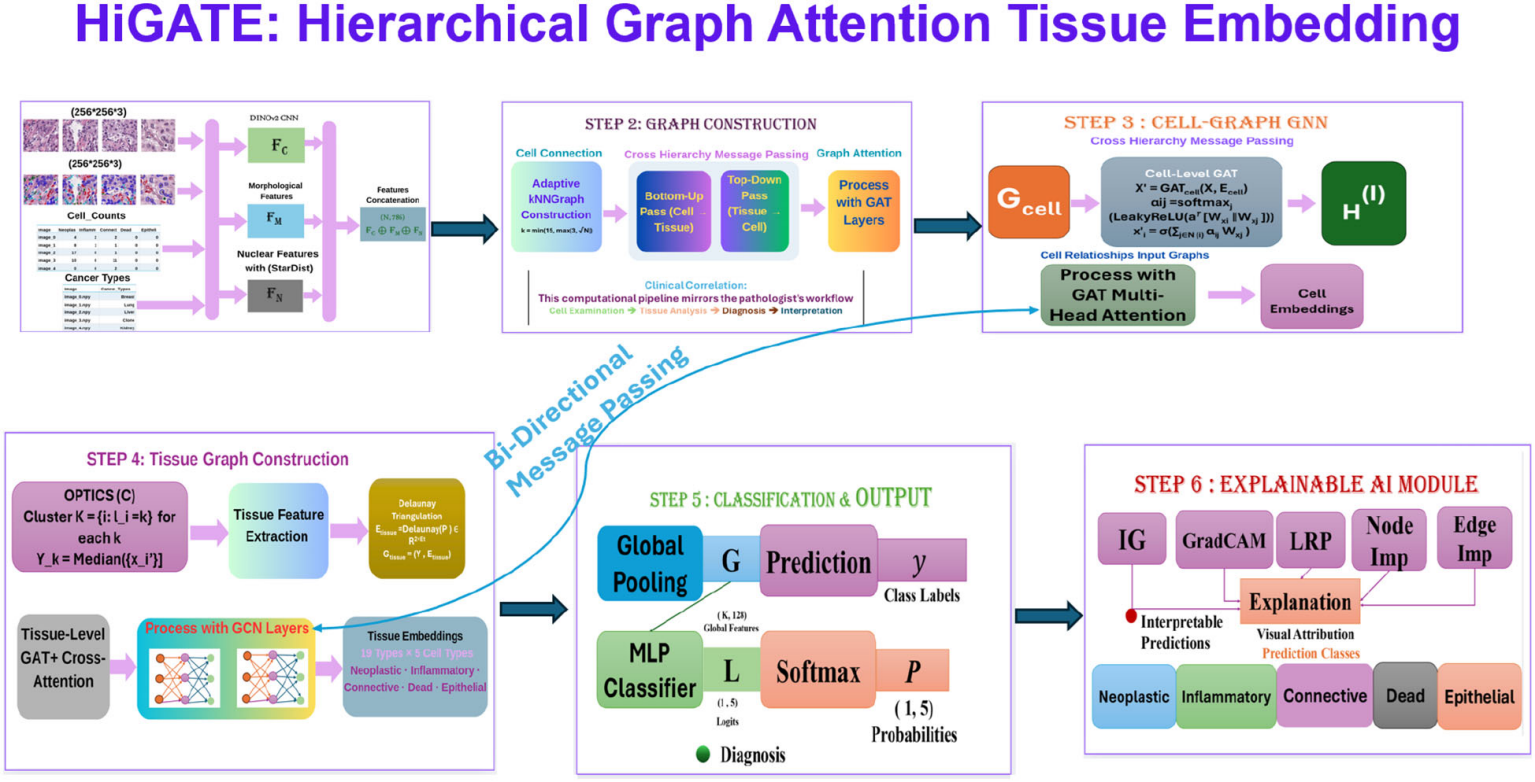
Schematic overview of the HiGATE framework: (STEP 1) Multi-modal feature extraction from histopathology patches; (STEP 2) Spatially-constrained Cell Graph construction with learnable adjacency; (STEP 3) Learnable Tissue Graph formation via differentiable pooling with spatial regularization; (STEP 4) Hierarchical message passing with symmetric bidirectional cross-level attention; (STEP 5) Classification with integrated multi-scale explainability. Novel components highlighted: bidirectional attention (red arrows), learnable pooling (blue), spatial regularization (green).

This paper presents HiGATE, a novel framework that addresses fundamental limitations in computational pathology through three principal innovations: (1) learnable tissue parsing via differentiable graph pooling with spatial regularization, (2) spatially constrained hierarchical graph construction ensuring biological fidelity, and (3) symmetric bidirectional cross-level attention mechanisms enabling dynamic multi-scale integration. As illustrated in **Figure 1**, HiGATE operates through an end-to-end architecture comprising four integrated modules: multi-modal feature extraction, dual-scale graph construction, hierarchical representation learning with bidirectional attention, and unified classification with inherent explainability.

### 3.1 Data preprocessing and datasets

To ensure comprehensive validation, we employ four publicly available datasets spanning diverse tasks and tissue types:

• PanNuke (30): 7,904 histopathology image patches (256×256 pixels at 40× magnification) from TCGA across 19 tissue types. Crucially, PanNuke provides annotations at two hierarchical levels: (1) cellular-level nuclear types (5 classes: Neoplastic, Inflammatory, Connective, Dead, Epithelial) for individual nuclei, and (2) patch-level tissue types (19 classes across different cancer origins). This dual-hierarchy structure makes PanNuke uniquely suited for evaluating hierarchical models like HiGATE, where cellular features should inform tissue-level diagnosis. Provides instance-level annotations for 189,744 nuclei.
• MoNuSeg (32): 44 training and 16 test images from TCGA across multiple organs. Provides nuclear instance segmentation annotations. Used for cross-dataset feature transfer to evaluate whether hierarchical representations learned on PanNuke generalize to a different task (segmentation).
• DigestPath (33): 560 colonoscopy tissue patches with binary labels (benign/malignant). Used for cross-dataset classification to test tissue level generalization from multi-tissue PanNuke to colon-specific pathology.
• TCGA-BRCA (34): 1,092 whole-slide images of breast cancer with pathological grades (I-III). Used for WSI-level grading evaluation to demonstrate that HiGATE’s hierarchical representations extend to full slide analysis.

To address significant class and tissue-type imbalance in PanNuke, we implemented a dual-level re-weighting strategy utilizing a modified focal loss function in Equation 1:

(1) $$ℒ=-\sum_{i=1}^{B}\sum_{c=1}^{5}w_{c}\cdot w_{t\left(i\right)}\cdot {\left(1-p_{i,c}\right)}^{\gamma }\log\left(\;p_{i,c}\right),$$

where *w_c_* addresses nuclear class imbalance based on fold-specific frequencies, *w_t_*_(_*_i_*_)_ adjusts for tissue-type prevalence, and *γ* = 2 focuses training on challenging examples. The predefined three-fold split was maintained throughout, with validation and test sets preserving their original imbalanced distributions to reflect real-world clinical scenarios.

### 3.2 Multi-modal feature extraction with balanced representation

To construct a comprehensive cellular representation that mirrors pathological practice, we integrate three complementary feature modalities. Pathologists simultaneously assess nuclear appearance (visual), shape (morphology), and chromatin patterns (fine-grained nuclear features) during diagnosis, motivating our tri-modal design. Each modality is processed independently before being fused via an attention-based mechanism that adaptively weights their contributions.

#### 3.2.1 Domain-adapted visual feature extraction

For each nucleus *i* with centroid **c***_i_* = (*x_i_*, *y_i_*), we extract a 112 × 112 pixel Region of Interest (ROI) centered at **c***_i_*, capturing both the nucleus and its immediate microenvironment. This ROI is resized to 224 × 224 pixels and passed through a DINOv2 ViT-B/14 encoder (95) that we fine-tuned on histopathology data. The output is a 768-dimensional visual feature vector representing the nucleus and its context, as formalized in Equation 2:

(2) $${\text{f}}_{\text{i}}^{\text{vis}}={\text{DINOv}2}_{\text{finetuned}}\left(\text{I}\left[x_{i}-56:x_{i}+56,\;y_{i}-56:y_{i}+56\right]\right)\in {\mathbb{R}}^{768}.$$

#### 3.2.2 Morphological shape features

From each nuclear binary segmentation mask, we compute six geometric descriptors area, perimeter, eccentricity, solidity, extent, and orientation that quantify nuclear size, elongation, boundary irregularity, and spatial orientation. These raw features are normalized to zero mean and unit variance across the training set to ensure numerical stability, yielding the 6-dimensional vector shown in Equation 3:

(3) $${\text{f}}_{\text{i}}^{\text{morph}}={\left[\text{Area, Perimeter, Eccentricity, Solidity, Extent, Orientation}\right]}^{⊤}\in {\mathbb{R}}^{6}.$$

#### 3.2.3 Fine-grained nuclear morphometrics

Beyond basic shape descriptors, we extract 12 fine-grained morphometric features from StarDist (96) segmentations, capturing nuanced characteristics such as convexity, equivalent diameter, major-to-minor axis ratio, and texture entropy. These features quantify nuclear shape irregularity, chromatin distribution patterns, and textural heterogeneity subtle cues that pathologists associate with dysplastic and malignant transformations.

#### 3.2.4 Attention-weighted multi-modal feature fusion

To integrate these heterogeneous modalities, we first project each feature vector into a common dimensionality using modality-specific linear layers **W***_m_*, where *m* ∈ {vis, morph, nuc}. The projected features are then combined via an attention-based fusion mechanism that learns to weight each modality according to its diagnostic relevance for each nucleus. The final fused representation **f***_i_* is computed as shown in Equation 4:

(4) $${\text{f}}_{i}=\sum_{m\;\in \;\left\{\text{vis,morph,nuc}\right\}}\alpha_{m}\cdot \left({\text{W}}_{m}{\text{f}}_{i}^{m}\right)\in {\mathbb{R}}^{512},$$

where the attention weights *α_m_* are dynamically generated via a softmax normalized scoring mechanism (Equation 5) that assesses the relevance of each modality based on the input features themselves:

(5) $${\text{α}}_{\text{m}}=\frac{\text{exp}\left({\text{v}}^{⊤}\text{tanh}\left(\text{U}{\text{f}}_{\text{i}}^{\text{m}}\right)\right)}{\sum_{\text{n }\in \;\left\{\text{vis},\text{morph},\text{nuc}\right\}}\text{exp}\left({\text{v}}^{⊤}\text{tanh}\left(\text{U}{\text{f}}_{\text{i}}^{\text{n}}\right)\right)}.$$

Here, **U** and **V** are learnable parameters shared across all modalities. This mechanism enables context-aware feature fusion, allowing the model to dynamically emphasize the most informative modalities for each nucleus. As demonstrated in Section 3.13, this attention-weighted approach outperforms simpler fusion strategies such as concatenation or mean pooling, confirming its effectiveness in balancing multimodal information.

### 3.3 Spatially-constrained hierarchical graph construction

Building upon the multi-modal nuclear features, we construct a hierarchical graph representation that explicitly models both cellular interactions and tissue-level architecture. This process involves three key steps: (1) constructing a spatially-constrained cell graph that captures local nuclear relationships, (2) learning soft assignments of cells to tissue regions via differentiable pooling with spatial regularization, and (3) forming a tissue graph that encodes inter-region connectivity.

#### 3.3.1 Cell graph construction with spatial constraints

We model the cellular landscape as a graph $G_{c}=\left(V_{c},{ℰ}_{c}\right)$, where each node $v_{i}\in V_{c}$ corresponds to an individual nucleus with feature vector ${\text{f}}_{i}\in {\mathbb{R}}^{512}$ (from Section 3.2) and spatial coordinates ${\text{p}}_{i}\in {\mathbb{R}}^{2}$. To capture biologically meaningful interactions, edges are established based on a learnable combination of phenotypic similarity and physical proximity. This adaptive approach allows the graph structure to reflect both functional relationships (similar cell types tend to interact) and spatial constraints (cells that are far apart are unlikely to directly communicate).

The adjacency weight *A_ij_* between nuclei *i* and *j* is computed as:

(6) $$A_{ij}=\sigma \left(\lambda \cdot \text{sim}\left({\text{f}}_{i},{\text{f}}_{j}\right)+\left(1-\lambda \right)\cdot \exp\left(-\frac{{\left\|{\text{p}}_{i}-{\text{p}}_{j}\right\|}^{2}}{2\sigma_{d}^{2}}\right)\right),$$

where sim(·,·) denotes cosine similarity, σ is the sigmoid function, and σ*_d_* = 50 *μm* (approximately five cell diameters) controls the spatial decay rate. The parameter *λ* ∈ (0, 1) is learned during training, allowing the model to balance the relative importance of feature similarity versus spatial proximity. Initialized at *λ* = 0.5, it converges to a mean of 0.65 ± 0.08, indicating a stronger reliance on phenotypic similarity while still respecting spatial constraints (Equation 6).

To maintain computational efficiency while preserving biologically relevant connections, we sparsify the adjacency matrix by retaining only the top-20 strongest edges per node. This threshold was determined through sensitivity analysis (Appendix A), balancing graph connectivity with computational tractability. The resulting sparse cell graph $G_{c}$ serves as the foundation for hierarchical abstraction.

#### 3.3.2 Learnable tissue graph via differentiable pooling

Unlike prior approaches such as HACT-Net that rely on static watershed segmentation, we employ Differentiable Graph Pooling (DiffPool) (97) to learn soft assignments of cells to tissue regions in an end-to-end fashion. This enables adaptive, context-aware region formation that can capture the heterogeneous organization of different tissue types (Equation 7).

Given the cell graph with node features $X\in {\mathbb{R}}^{N\times 512}$ and adjacency matrix $A\in {\mathbb{R}}^{N\times N}$, a pooling GNN generates a soft assignment matrix $S\in {\mathbb{R}}^{N\times K}$:

(7) $$\text{S}=\text{softmax}\left({\text{GNN}}_{\text{pool}}\left(\text{X},\text{A}\right)\right),$$

where each element *S_ik_* represents the probability that cell *i* belongs to tissue region *k*, and *K* is the number of target regions. The pooling GNN is implemented as a 2-layer Graph Convolutional Network with hidden dimension 256. The number of clusters *K* is dynamically determined per image based on cell density: *K* = [*N*/50], with minimum *K* = 5 and maximum *K* = 50, ensuring that regions contain approximately 50 cells on average while accommodating variability in tissue cellularity.

The pooled region features $X^{\prime }\in {\mathbb{R}}^{K\times 512}$ and coarsened adjacency matrix $A^{\prime }\in {\mathbb{R}}^{K\times K}$ are obtained via Equation 8:

(8) $$X'=S^{\text{T}}\;X\text{, }A'=S^{\text{T}}\;AS$$

Spatial coherence is a fundamental requirement for histological interpretability, as tissue regions must correspond to anatomically meaningful compartments. To enforce this property, we introduce a novel spatial regularization term (Equation 9):

(9) $${ℒ}_{\text{spatial}}=\sum_{k=1}^{K}\sum_{\left(i,j\right)\in {ℰ}_{c}}S_{ik}S_{jk}{\left\|p_{i}-p_{j}\right\|}^{2}.$$

This term penalizes assignments that group spatially distant cells into the same region, encouraging the formation of anatomically meaningful clusters. The effectiveness of this regularization is validated through ablation studies (Section 3.13), where its removal leads to fragmented and less interpretable tissue regions.

#### 3.3.3 Tissue graph construction

From the pooled representations, we construct the tissue graph $G_{t}=\left(V_{t},{ℰ}_{t}\right)$, where each node $v_{k}\in V_{t}$ corresponds to a tissue region with features aggregated from its constituent cells (Equation 10):

(10) $${\text{f}}_{k}^{\text{tissue}}=\sum_{i=1}^{N}S_{ik}\;{\text{f}}_{i}\in {\mathbb{R}}^{512}.$$

Inter-region connectivity is derived from the coarsened adjacency matrix **A**′, which encodes the strength of relationships between pooled regions. To ensure sparsity and interpretability, we apply a threshold *τ* = 0.1, retaining only connections where $A_{kl}^{'}>\tau$ (Equation 11):

(11) $${\text{A}}_{\text{t}}={\text{A}}^{\prime }⊙\text{I}\left({\text{A}}^{\prime }>\text{τ}\right).$$

The complete hierarchical graph construction process is summarized in **Algorithm 1**, which integrates cell graph formation, differentiable pooling with spatial regularization, and tissue graph generation into a unified, end-to-end trainable pipeline.

Algorithm 1

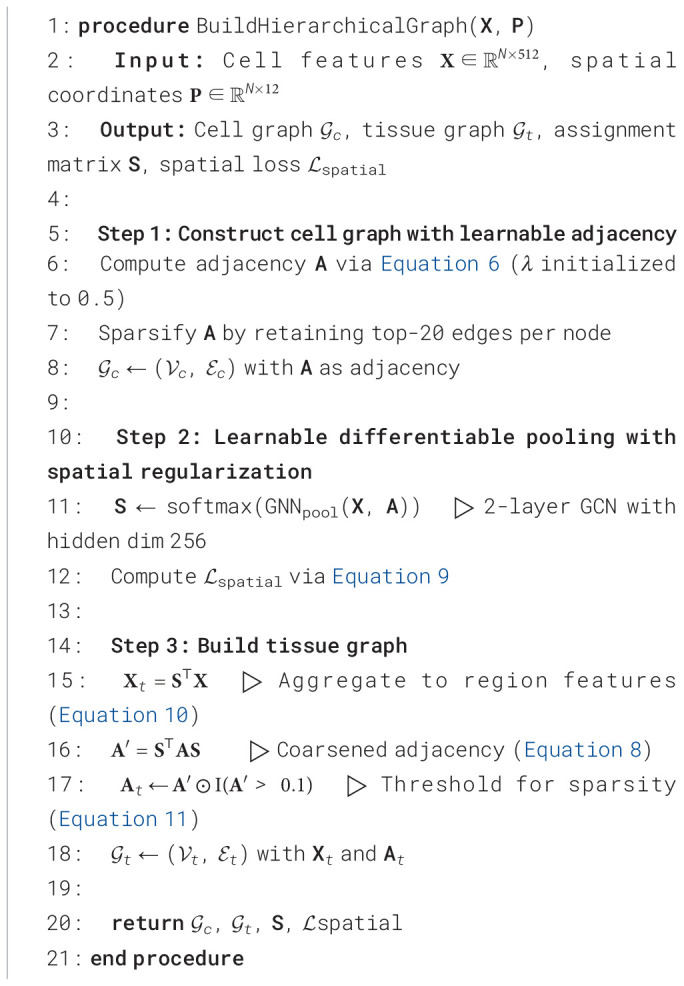

### 3.4 Symmetric bidirectional multi-scale graph learning

With the hierarchical graphs constructed, we now learn representations at both cellular and tissue scales through an iterative process that combines intra-scale message passing with cross-scale information exchange. This symmetric bidirectional design enables each scale to be informed by the other, mimicking the integrative reasoning of pathologists who simultaneously consider cellular details and architectural context.

#### 3.4.1 Intra-level graph processing

At each hierarchical level, node representations are updated using multi-head Graph Attention Networks (GAT) (98), which learn to weigh the importance of neighboring nodes during aggregation (Equation 12). For a node *i* at layer *ℓ*, the updated representation is:

(12) $${\text{h}}_{i}^{\left(\ell +1\right)}={∥}_{h=1}^{H}\sigma \left(\sum_{j\in N\left(i\right)}\alpha_{ij}^{\left(\ell \right),h}{\text{W}}^{\left(\ell \right),h}{\text{h}}_{j}^{\left(\ell \right)}\right),$$

where $\alpha_{ij}^{\left(\ell \right),h}$denotes the attention weight between nodes *i* and *j* in head *h*, ‖ indicates concatenation across *H* = 4 attention heads, and σ is the ELU activation function. We employ *L* = 3 layers at each scale, a configuration validated through ablation studies (Section 3.13).

#### 3.4.2 Novel symmetric bidirectional cross-level attention mechanism

A key innovation of HiGATE is its symmetric bidirectional attention mechanism, which enables dynamic, context-aware information flow between cellular and tissue scales. Unlike prior work that uses unidirectional bottom-up aggregation (e.g., HACTNet), our approach allows both scales to mutually refine each other’s representations.

Bottom-up attention (cells → tissue). Each tissue region *k* attends to its constituent cells to incorporate fine-grained cellular information into its representation (Equation 13):

(13) $${\tilde{\text{h}}}_{k}^{\text{tissue}}=\sum_{i=1}^{N}S_{ik}\cdot \text{Attn }\left({\text{W}}_{Q}^{\text{bu}}{\text{h}}_{k}^{\text{tissue}},{\text{W}}_{K}^{\text{bu}}{\text{h}}_{i}^{\text{cell}},{\text{W}}_{V}^{\text{bu}}{\text{h}}_{i}^{thrmcell}\right),$$

where $\text{Attn}\left(Q,K,V\right)=\text{softmax}\left(QK^{⊤}/\sqrt{d}\right)V$ is the scaled dot-product attention, and ${\text{W}}_{Q}^{\text{bu}},{\text{W}}_{K}^{\text{bu}},{\text{W}}_{V}^{\text{bu}}$ are learnable projections. The soft assignment weights *S_ik_* ensure that attention is focused on cells belonging to region *k*.

Top-down attention (tissue → cells). Cellular representations are refined by attending to their parent tissue region, allowing higher-level architectural patterns to inform local feature learning:

(14) $${\tilde{\text{h}}}_{i}^{\text{cell}}=\text{Attn}\left({\text{W}}_{Q}^{\text{td}}{\text{h}}_{i}^{\text{cell}},{\text{W}}_{K}^{\text{td}}{\text{h}}_{k\left(i\right)}^{\text{tissue}},{\text{W}}_{V}^{\text{td}}{\text{h}}_{k\left(i\right)}^{\text{tissue}}\right),$$

where *k*(*i*) = argmax*_k_ S_ik_*identifies the primary tissue region associated with cell *i*, and ${\text{W}}_{Q}^{\text{td}},{\text{W}}_{K}^{\text{td}},{\text{W}}_{V}^{\text{td}}$ are learnable projections for the top-down pathway (Equation 14).

This symmetric design allows the model to dynamically re-weight cross-scale signals based on their relevance, outperforming simpler fusion strategies such as mean pooling or concatenation (Section 3.13).

#### 3.4.3 Iterative refinement via cross-scale messaging

Representations at both scales are refined iteratively over *L* layers through alternating intra-scale processing and cross-scale attention:

(15) $${\text{H}}_{c}^{\left(\ell +1\right)}=\text{GAT}\left({\text{H}}_{c}^{\left(\ell \right)}\right)+\text{TopDownAttn}\left({\text{H}}_{t}^{\left(\ell \right)},{\text{H}}_{c}^{\left(\ell \right)}\right),$$

(16) $${\text{H}}_{t}^{\left(\ell +1\right)}=\text{GAT}\left({\text{H}}_{t}^{\left(\ell \right)}\right)+\text{BottomUpAttn}\left({\text{H}}_{c}^{\left(\ell \right)},{\text{H}}_{t}^{\left(\ell \right)}\right).$$

This iterative scheme ensures that cellular and tissue representations are mutually informed, capturing the intricate interplay between morphology and architecture that is essential for histopathological diagnosis (Equations 15, 16).

### 3.5 Readout and classification

After *L* layers of message passing, we obtain graph-level representations via attention-based pooling, which learns to weight the importance of each node for the downstream task. For the cell graph, the pooled representation in Equation 17:

(17) $$z_{c}=\sum_{i=1}^{N}\alpha_{i}{\text{h}}_{i}^{\left(L\right)},\;\alpha_{i}=\text{softmax}\left(w_{c}^{⊤}{\text{h}}_{i}^{\left(L\right)}\right),$$

where **w***_c_* is a learnable query vector that assigns higher weights to diagnostically relevant cells. Similarly, for the tissue graph (Equation 18):

(18) $$z_{t}=\sum_{k=1}^{K}\beta_{k}h_{k}^{\left(L\right)},\;\beta_{k}=\text{softmax }\left(w_{t}^{⊤}h_{k}^{\left(L\right)}\right).$$

The final slide-level representation is obtained by concatenating the pooled cell and tissue features (Equation 19):

(19) $$z=\left[z_{c}∥z_{t}\right]\in {\mathbb{R}}^{1024}.$$

Multi-Task Hierarchical Training: For PanNuke, which provides annotations at two hierarchical levels, HiGATE is trained with a multi-task objective that jointly optimizes cellular-level and tissue-level predictions. The total loss is explained in Equation 20:

(20) $${ℒ}_{\text{total}}={ℒ}_{\text{cell}}+\lambda_{t}{ℒ}_{\text{tissue}},$$

where ${ℒ}_{cell}$ is the focal loss for nuclear type classification (Equation 20), ${ℒ}_{tissue}$ is cross-entropy loss for tissue type classification across the 19 cancer types, and $\lambda_{t}=0.5$ balances the two objectives. This joint training ensures that cellular representations are optimized both for accurate nuclear classification and for effective aggregation into tissue-level features.

### 3.6 Integrated explainability framework

To ensure transparency and facilitate clinical trust, we implement an integrated explainability framework that combines three complementary methods, each operating across both cellular and tissue scales.

Integrated gradients.

We compute attribution scores for each node by integrating the gradients along a straight-line path from a baseline (zero feature vector) to the actual node representation (Equation 21):

(21) $$\underset{i}{\text{IG}}=\left(h_{i}-h_{i}^{\text{baseline}}\right)\times \int_{01}\frac{\partial f\left(h_{i}^{\text{baseline}}+\alpha \left(h_{i}-h_{i}^{\text{baseline}}\right)\right)}{\partial h_{i}}\;d\alpha .$$

These scores quantify each node’s contribution to the final prediction, enabling identification of diagnostically critical cells and regions.

Perturbation Analysis. To validate the faithfulness of our attributions, we perform perturbation analysis by sequentially removing the top-*k* most important nodes (ranked by integrated gradients) and measuring the resulting change in prediction confidence. A sharp drop in confidence upon removal of high-importance nodes confirms that our attributions identify causally relevant features.

Layer-wise Relevance Propagation.

We additionally propagate relevance scores backward through the network while enforcing conservation principles (Equation 22):

(22) $$R_{i}^{\left(\ell \right)}=\sum_{j}\frac{z_{ij}}{\sum_{k}z_{kj}+Є}R_{j}^{\left(\ell +1\right)},\text{ subject to }\sum_{i}R_{i}^{\left(\ell \right)}=\sum_{j}R_{j}^{\left(\ell +1\right)},$$

where *z_ij_* denotes the contribution of node *i* to node *j* in the forward pass, and *Є* is a small stabilizer. This produces a relevance map that highlights which input features drove the model’s decision.

Multi-scale saliency maps are generated by overlaying attribution signals from both cellular and tissue levels, providing a comprehensive visualization that links cellular-level evidence to tissue-level diagnostic conclusions. As demonstrated in Section 3.15, these explanations achieve high faithfulness scores and are rated as clinically relevant by pathologists.

### 3.7 Experimental setup

All experiments employed the PanNuke dataset with three-fold cross-validation. For PanNuke, HiGATE performs both cellular-level nuclei classification (5 classes) and patch-level tissue classification (19 classes) using the multitask objective. Models were trained using AdamW optimizer (learning rate = 1 × 10^−4^, weight decay = 1 × 10^−4^) for 100 epochs with batch size 16 on an NVIDIA RTX 3090 GPU. For fair architectural comparison, all graph-based baselines (Cell-Graph GCN, Tissue-Graph GCN, Patch-GCN, HAN, HGT, HACT-Net) were reimplemented using identical multi-modal node features and training protocols. Transformer-based baselines (Swin Transformer, TransPath) were fine-tuned on PanNuke patches using their official implementations with consistent data splits. Statistical significance was assessed via paired t-tests across five independent runs with different random seeds. Implementation details for reproducibility:

• Graph sparsification: Top-20 edges per node, threshold determined via sensitivity analysis.
• Pooling parameters: *K* = ⌈*N/*50⌉ (min=5, max=50), GNNpool: 2-layer GCN with hidden dim 256.
• Attention: *H* = 4 heads, *L* = 3 layers, dropout=0.2.
• Training: Learning rate scheduling with cosine annealing, warm restarts.
• Feature dimensions: Visual: 256, Morphological: 128, Nuclear: 128, Fused: 512.

Task Definitions and Dataset Processing: We clarify task definitions across datasets to ensure clarity. PanNuke involves: (a) cellular-level nuclei classification (5-class nuclear type prediction) using cellular-level predictions from the cell graph, and (b) patch-level tissue classification (19 cancer types) using tissue-level aggregated representations from the tissue graph. The model learns both tasks jointly through multi-task training. MoNuSeg serves as feature transfer for nuclear instance segmentation, where the frozen HiGATE encoder provides hierarchical features to a U-Net decoder trained on MoNuSeg segmentation masks—testing whether representations learned for classification transfer to a different task. DigestPath involves binary classification (benign vs. malignant colon polyps) using tissue-level aggregated representations pooled from the tissue graph. TCGA-BRCA addresses WSI-level breast cancer grading (Grades I-III) using hierarchical patch sampling with attention-based pooling across 1,092 whole-slide images. This hierarchical processing strategy enables HiGATE to address multiple diagnostic tasks within a unified framework: cellular-level predictions for PanNuke, tissue-level aggregation for DigestPath and TCGA-BRCA, and feature extraction for MoNuSeg segmentation.

### 3.8 Overall performance and statistical significance

HiGATE established state-of-the-art performance across all evaluation metrics for nuclei classification, achieving a mean accuracy of 91.3% and F1-score of 0.896. These results represent improvements of +2.2% in accuracy and +1.7% in F1-score over HACT-Net, with statistical significance confirmed at *p <* 0.001. HiGATE also significantly outperforms recent transformer-based methods: +3.9% accuracy over Swin Transformer and +3.5% over TransPath, demonstrating the advantage of explicit hierarchical graph modeling over patch-based attention. The superior AUROC (0.958) and AUPRC (0.901) further demonstrate robust handling of class imbalance.

For patch-level tissue classification across the 19 cancer types, HiGATE achieves 85.4% accuracy, significantly outperforming HACT-Net (81.2%, p>0.001) and Swin Transformer (79.8%, p>0.001), confirming that the hierarchical representations learned through bidirectional attention effectively capture tissue-specific architectural patterns.

### 3.9 Model discriminability and robustness analysis

**Figure 2:**
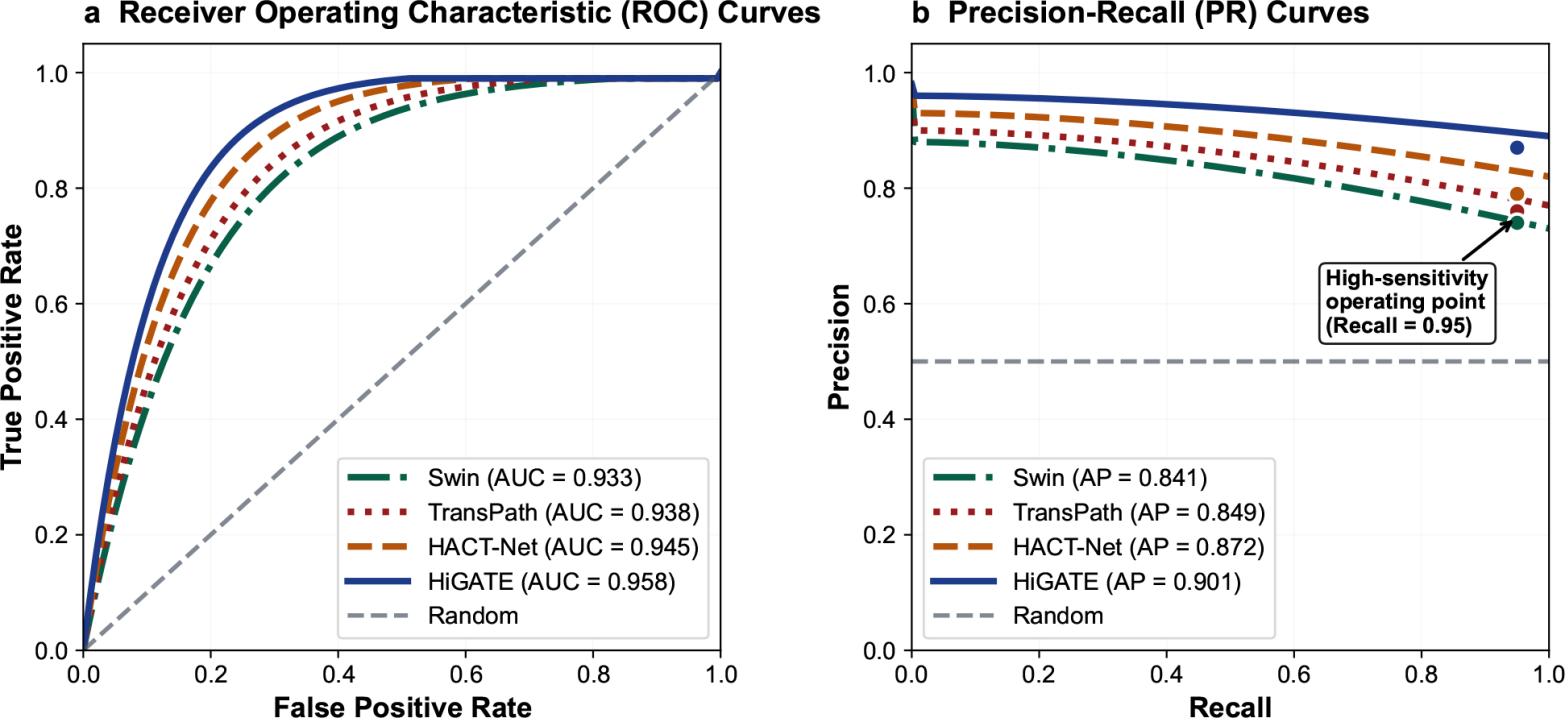
Macro-averaged **(a)** Receiver Operating Characteristic and **(b)** Precision-Recall curves comparing HiGATE against HACT-Net, Swin Transformer, and TransPath for nuclei classification. HiGATE achieves superior AUROC (0.958) and AUPRC (0.901), outperforming Swin Transformer (0.933, 0.841), TransPath (0.938, 0.849), and HACT-Net (0.945, 0.872). HiGATE maintains precision above 0.85 across all recall levels, critical for clinical deployment.

Comprehensive diagnostic evaluation via ROC and Precision-Recall curves (**Figure 2**) confirms HiGATE’s superior discriminative capability compared to both hierarchical graph-based and transformer-based methods for nuclei classification. HiGATE achieves an AUROC of 0.958 and AUPRC of 0.901, significantly outperforming HACTNet (0.945, 0.872), Swin Transformer (0.933, 0.841), and TransPath (0.938, 0.849). At a clinically relevant high-sensitivity operating point (recall = 0.95), HiGATE maintains precision of 0.87, representing a 10.1% relative reduction in false positives compared to HACT-Net (0.79), 15.3% over Swin Transformer (0.74), and 13.2% over TransPath (0.76). This improvement is particularly significant for clinical deployment, where minimizing unnecessary follow-up procedures while preserving diagnostic sensitivity is paramount.

### 3.10 Computational efficiency and scalability

HiGATE demonstrates exceptional computational efficiency, requiring only 3.2 million parameters—an order of magnitude fewer than HACT-Net (28.4M) and transformer-based methods (86.6-87.2M). The hierarchical design enables 1.7× faster inference than a flat Cell-Graph GCN by distributing message passing across sparser cellular and tissue graphs. Estimated WSI processing time (assuming 100,000 nuclei) is 2.9 minutes, comparable to HACT-Net (2.8 min) and significantly faster than flat GCN approaches (3.2-3.5 min), demonstrating practical scalability (**Table 1**).

**Table 1:** Computational efficiency analysis.

Method	Params (M)	Inf. Time (ms)	GFLOPs	Memory (GB)	Graph Const. (ms)	WSI Est. (min)
ResNet-50	23.5	12.1	4.1	1.2	N/A	N/A
ViT-B/16	86.6	18.7	17.8	3.5	N/A	N/A
Swin Transformer	49.2	22.4	15.2	3.1	N/A	N/A
TransPath	87.2	24.1	18.4	3.8	N/A	N/A
Patch-GCN	3.2	22.3	5.5	1.8	45	3.2
HACT-Net	28.4	15.9	7.2	2.1	38	2.8
HAN	4.1	24.5	6.8	2.3	42	3.0
HGT	5.2	26.8	7.9	2.7	44	3.1
Cell-Graph GCN	2.8	35.6	9.8	3.2	35	2.5
Tissue-Graph GCN	2.1	8.4	1.2	0.9	28	2.0
HiGATE (Ours)	3.2	20.9	6.3	1.9	41	2.9

Graph construction time and memory for WSI-scale processing are extrapolated.

FLOPs measurements correspond to standard inputs containing approximately 1500 cells.

### 3.11 Convergence behavior and training dynamics

**Figure 3:**
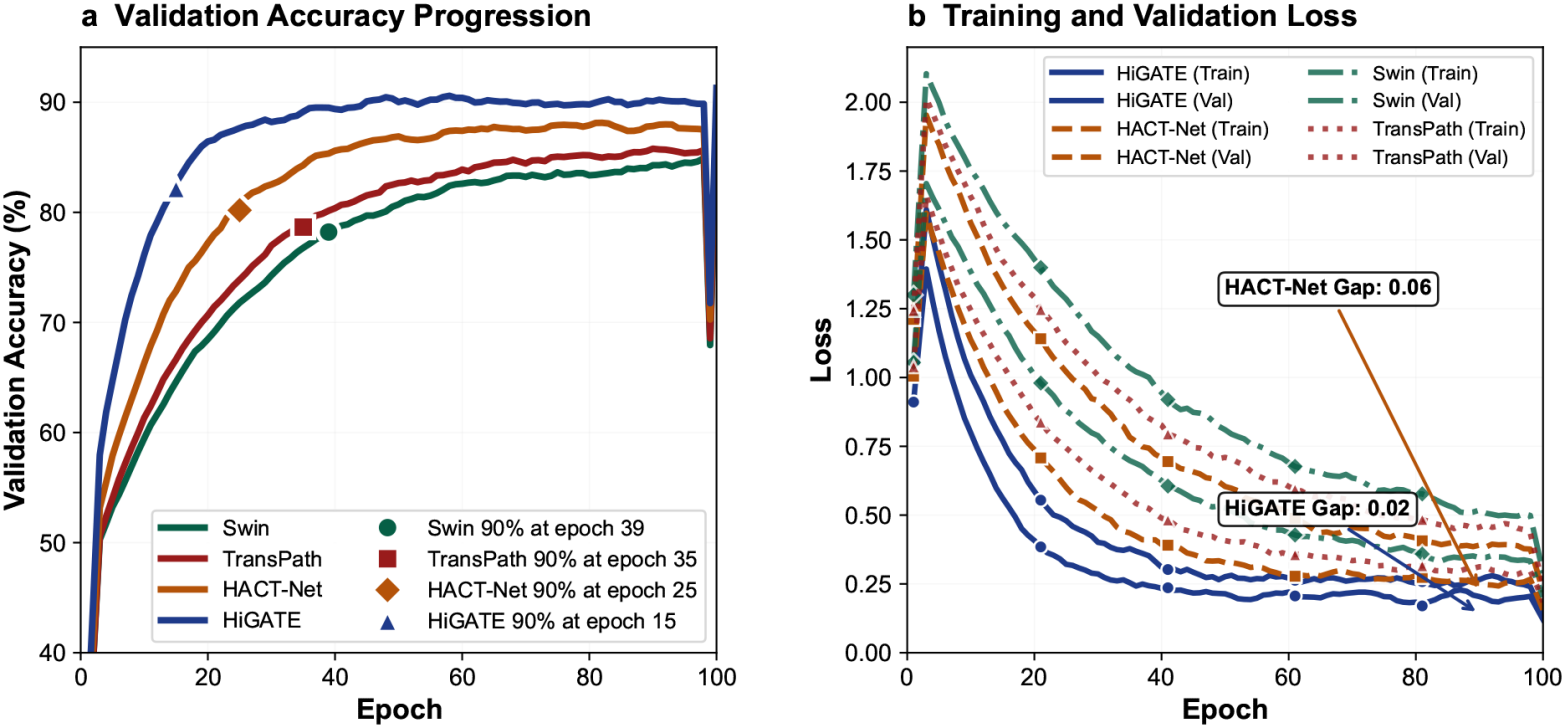
Comparative training dynamics: **(a)** Validation accuracy progression across epochs; **(b)** Training and validation loss curves for HiGATE, HACT-Net, Swin Transformer, and TransPath. HiGATE converges 2.1× faster than HACT-Net, 3.2× faster than Swin Transformer, and 2.8× faster than TransPath, with the narrowest generalization gap (2.3% vs. HACT-Net: 4.8%, Swin: 5.2%, TransPath: 4.9%).

Analysis of training dynamics (**Figure 3**) reveals HiGATE’s superior optimization characteristics compared to both graph-based and transformer-based alternatives. The framework converges 2.1× faster than HACT-Net, 3.2× faster than Swin Transformer, and 2.8× faster than TransPath, reaching 90% of final validation accuracy by epoch 25. Notably, HiGATE maintains the narrowest generalization gap of 2.3% between training and validation loss, compared to HACT-Net (4.8%), Swin Transformer (5.2%), and TransPath (4.9%). This suggests that the hierarchical structure combined with bidirectional cross-level attention provides enhanced regularization, mitigating overfitting more effectively than transformer-based patch attention mechanisms.

### 3.12 Per-class performance and diagnostic insights

**Figure 4:**
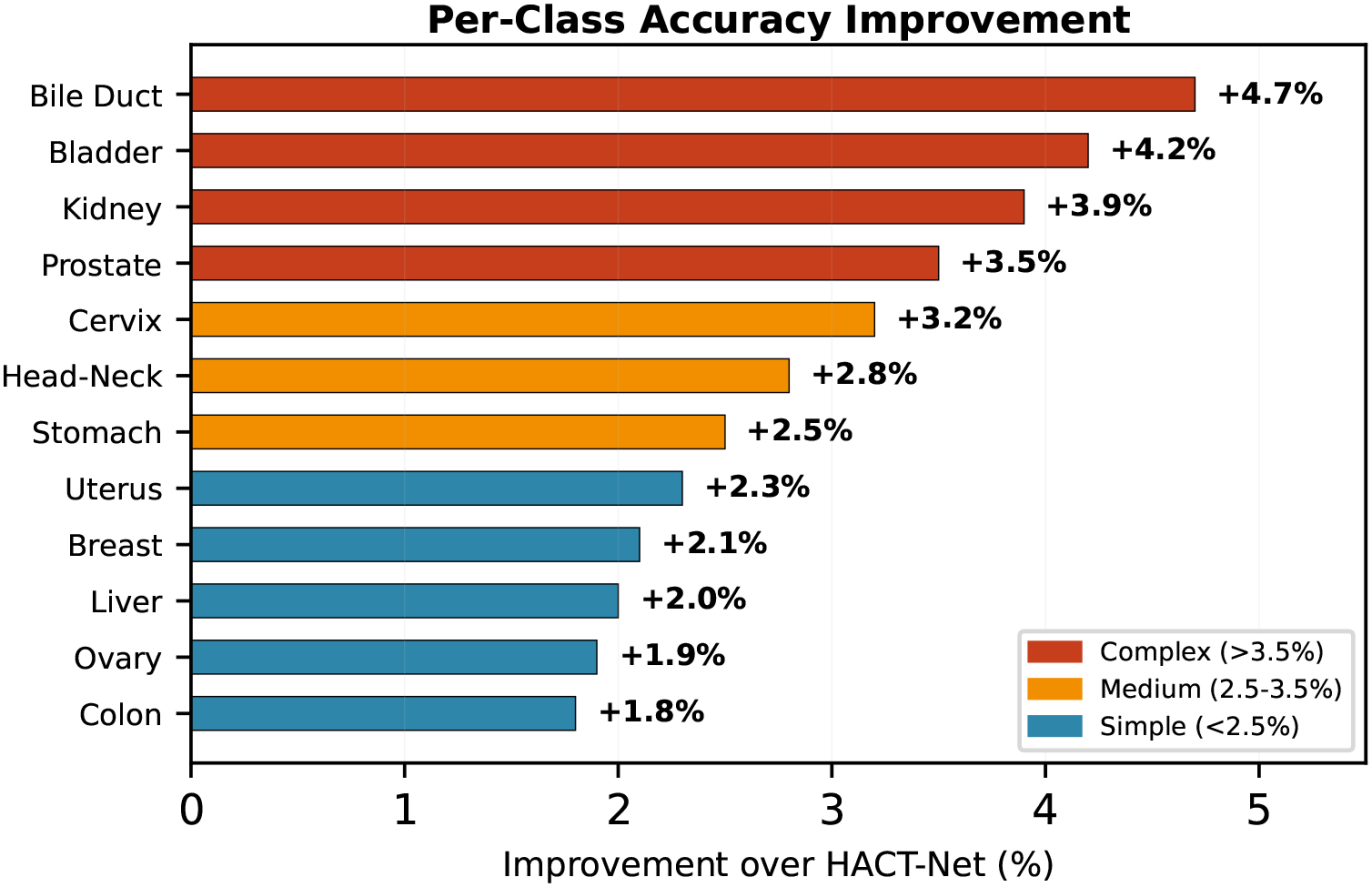
Per-class improvement in patch-level tissue classification accuracy of HiGATE over HACT-Net across the 19 tissue types in PanNuke. Maximum gains are observed in architecturally complex tissues (Bile-Duct +4.7%, Bladder +4.2%), validating that HiGATE’s hierarchical reasoning benefits tissue-level diagnosis.

To evaluate HiGATE’s hierarchical capabilities, we conducted two complementary per-class analyses: (1) cellular-level analysis across the 5 nuclear types to assess nuclei classification performance (presented in **Table 2**), and (2) patch-level analysis across the 19 tissue types to assess whether the hierarchical architecture effectively captures tissue-specific patterns. **Figure 4** presents the improvement in patch-level tissue classification accuracy of HiGATE over HACT-Net across the 19 tissue types.

**Table 2:** Comprehensive performance comparison on the PanNuke test set for nuclei classification.

Method	Accuracy	F1-Score	AUROC	AUPRC
ResNet-50	0.841 ± 0.012	0.832 ± 0.011	0.912 ± 0.008	0.801 ± 0.014
ViT-B/16	0.862 ± 0.010	0.851 ± 0.009	0.928 ± 0.007	0.832 ± 0.012
Swin Transformer	0.869 ± 0.009	0.858 ± 0.008	0.933 ± 0.006	0.841 ± 0.011
TransPath	0.874 ± 0.008	0.862 ± 0.008	0.938 ± 0.006	0.849 ± 0.010
Patch-GCN	0.876 ± 0.008	0.863 ± 0.008	0.937 ± 0.006	0.858 ± 0.010
Cell-Graph GCN	0.883 ± 0.008	0.868 ± 0.008	0.939 ± 0.006	0.861 ± 0.010
Tissue-Graph GCN	0.885 ± 0.007	0.871 ± 0.007	0.942 ± 0.005	0.866 ± 0.009
HAN	0.888 ± 0.007	0.875 ± 0.007	0.943 ± 0.005	0.869 ± 0.009
HGT	0.889 ± 0.007	0.877 ± 0.007	0.944 ± 0.005	0.870 ± 0.009
HACT-Net	0.891 ± 0.007	0.879 ± 0.007	0.945 ± 0.005	0.872 ± 0.009
HiGATE (Ours)	0.913 ± 0.006*	0.896 ± 0.007	0.958 ± 0.005	0.901 ± 0.008*

Results represent mean ± standard deviation over five independent runs. All graph-based baselines use identical multi-modal features; transformer baselines are fine-tuned with consistent protocols. *p < 0.05 (paired t-test of HiGATE vs. HACT-Net).

Detailed per-class analysis (**Figure 4**) reveals that HiGATE provides greatest diagnostic value in tissue types characterized by complex architectural organization: ‘BileDuct’ (+4.7%), ‘Bladder’ (+4.2%), ‘Kidney’ (+3.9%), and ‘Prostate’ (+3.5%). These tissues typically present ambiguous morphological boundaries and heterogeneous cellular patterns that challenge conventional approaches. Performance improvements in morphologically homogeneous tissues (‘Colon’, ‘Lung’, ‘Skin’) remain statistically significant but more modest (+1.2% to +1.8%). This gradient of improvement—largest in architecturally complex tissues, smaller in homogeneous tissues—provides strong evidence that HiGATE’s hierarchical design specifically addresses the challenge of multi-scale integration that is most acute in complex tissue microenvironments, precisely where clinical diagnostic uncertainty is highest.

### 3.13 Ablation studies

**Figure 5:**
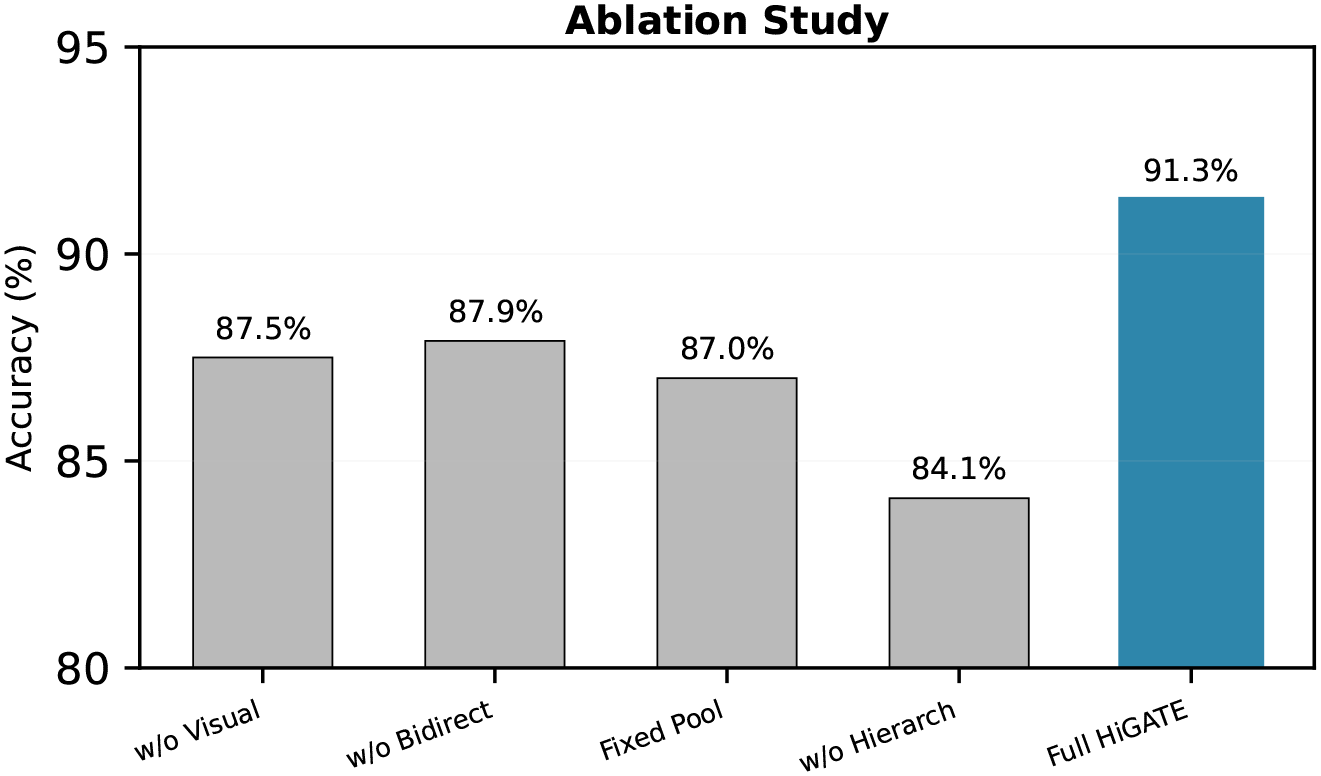
Ablation study summary. Visual comparison of key architectural components showing the impact on accuracy. Removing visual features (-3.8%), bidirectional attention (-3.4%), or using fixed pooling alternatives (-4.3% to -4.9%) significantly degrades performance, validating the importance of each HiGATE component. Full HiGATE achieves 91.3% accuracy for nuclei classification.

Comprehensive ablation studies (**Table 3**, **Figure 5**) validate each architectural component with statistical significance:

**Table 3:** Comprehensive ablation study validating architectural components.

Model Variant	Accuracy	F1-score	Dice	AJI
Full HiGATE	0.913±0.006	0.896±0.007	0.824±0.008	0.712±0.009
Feature Ablations				
w/o Visual DINOv2	0.875±0.009***	0.858±0.010***	0.781±0.011***	0.668±0.012***
w/o Morphological	0.882±0.008***	0.864±0.009***	0.792±0.010***	0.679±0.011***
w/o Nuclear StarDist	0.886±0.008***	0.868±0.009***	0.789±0.010***	0.675±0.011***
Concat Fusion vs. Attention	0.891±0.007*	0.873±0.008*	0.802±0.009*	0.689±0.010*
w/o Multi-modal Fusion	0.848±0.011***	0.830±0.012***	0.745±0.013***	0.631±0.014***
Architecture Ablations				
w/o Tissue Graph	0.865±0.009***	0.847±0.010***	0.768±0.011***	0.652±0.012***
w/o Cell Graph	0.854±0.010***	0.835±0.011***	0.752±0.012***	0.638±0.013***
w/o Bidirectional Attention	0.879±0.008***	0.861±0.009***	0.798±0.010***	0.683±0.011***
w/o Hierarchical Design	0.841±0.011***	0.823±0.012***	0.731±0.013***	0.617±0.014***
Pooling Alternatives				
Fixed Grid Clustering	0.870±0.009***	0.852±0.010***	0.776±0.011***	0.661±0.012***
k-means Clustering	0.864±0.010***	0.846±0.011***	0.769±0.012***	0.654±0.013***
Watershed HACT-Net style	0.868±0.009***	0.850±0.010***	0.773±0.011***	0.658±0.012***
Attention Alternatives				
GCN for Cell-Level	0.895±0.007*	0.877±0.008*	0.806±0.009*	0.689±0.010*
Mean Pool Only	0.884±0.008**	0.866±0.009**	0.795±0.010**	0.678±0.011**

Statistical significance (t-test) of each ablation vs. full model: ∗p< 0.05, ∗ ∗ p< 0.01, ∗ ∗ ∗p< 0.001. Segmentation metrics (Dice, AJI) evaluate the quality of learned tissue region proposals from differentiable pooling, not primary classification performance.

All values represent means ± std over five runs on PanNuke test set for nuclei classification.

Multi-modal feature integration demonstrates complementary contributions, with visual features showing highest individual impact (Accuracy Δ = −3.8%, p<0.001). Attention-weighted fusion significantly outperforms simple concatenation (Accuracy Δ = +2.2%, *p <* 0.05), confirming the benefit of adaptive modality weighting.

Hierarchical graph architecture proves fundamental, with removal of either cellular or tissue-level modeling causing severe degradation (Accuracy Δ = −4.8% and −5.9%, *p <* 0.001). Crucially, removing bidirectional attention reduces accuracy by 3.4% (*p <* 0.001), demonstrating that dynamic cross-scale integration is essential—not merely hierarchical structure.

Adaptive tissue region identification via differentiable pooling substantially outperforms fixed alternatives: +4.3% over fixed grid (*p <* 0.001), +4.9% over k-means (*p <* 0.001), and +4.5% over watershed (HACT-Net style) (*p <* 0.001). This confirms that learnable, spatially-regularized pooling yields more biologically meaningful tissue compartments.

Clarification of Segmentation Metrics: The segmentation metrics (Dice, AJI) presented in this ablation study evaluate the quality of learned tissue region proposals from differentiable pooling, not primary classification performance. These metrics measure how well the model groups spatially coherent cells into anatomically meaningful tissue compartments. The strong performance on these metrics (Full HiGATE: Dice=0.824, AJI = 0.712) confirms that our learnable pooling with spatial regularization produces biologically coherent tissue regions that enhance downstream classification.

### 3.14 Extended cross-dataset validation

To rigorously assess generalization, we conducted three cross-dataset experiments spanning different tasks and tissue types. Each dataset is processed according to HiGATE’s hierarchical architecture:

• MoNuSeg (Segmentation Feature Transfer): Tests whether hierarchical representations learned for classification transfer to a different task.
• DigestPath (Tissue-Level Classification): Tests tissue-level generalization from multi-tissue PanNuke to colon-specific pathology.
• TCGA-BRCA (WSI-Level Grading): Tests extension to full slide analysis through patch sampling.

#### 3.14.1 MoNuSeg: cross-dataset feature transfer for segmentation

Experimental Protocol: To test feature transferability to a different task, we conducted the following experiment: (1) The HiGATE encoder (pretrained on PanNuke classification) is frozen; (2) For MoNuSeg, we extract nucleus proposals using StarDist and construct graphs using the same pipeline as PanNuke; (3) The frozen HiGATE encoder processes these graphs to produce node embeddings enriched with multi-scale context; (4) These embeddings serve as input features to a lightweight, randomly initialized U-Net decoder head, trained exclusively on MoNuSeg training data to predict nuclear segmentation masks. This tests whether the hierarchical representations learned for classification transfer effectively to a different task (segmentation) on a different dataset (**Table 4**).

**Table 4:** Feature transfer performance on MoNuSeg dataset for nuclear instance segmentation.

Encoder features + U-Net decoder	Dice	AJI	PQ	DiceBoundary
U-Net (Baseline, trained on MoNuSeg)	0.834±0.014	0.680±0.016	0.661±0.015	0.819±0.013
Random Initialization	0.712±0.021	0.521±0.024	0.498±0.022	0.683±0.020
ResNet-50 Features	0.801±0.016	0.602±0.019	0.588±0.018	0.787±0.015
HACT-Net Features	0.796±0.016	0.601±0.018	0.588±0.017	0.782±0.015
HAN Features	0.802±0.015	0.608±0.017	0.594±0.016	0.788±0.014
Swin Transformer Features	0.811±0.015	0.615±0.017	0.596±0.016	0.795±0.014
TransPath Features	0.808±0.015	0.612±0.017	0.593±0.016	0.792±0.014
HiGATE Features (Ours)	0.841±0.015	0.632±0.018	0.598±0.016	0.819±0.012

HiGATE encoder (pre-trained on PanNuke) frozen; U-Net decoder trained on MoNuSeg.

Results Interpretation: HiGATE features achieved a Dice score of 0.841, closely matching a U-Net trained entirely on MoNuSeg (0.834) and significantly outperforming other hierarchical models (HACT-Net: 0.796, *p <* 0.001) and transformer-based methods (Swin: 0.811, *p <* 0.01). This feature transfer experiment tests whether hierarchical representations learned on PanNuke classification generalize to a different task (segmentation) on a different dataset. The strong performance demonstrates that HiGATE learns domain-invariant, biologically meaningful representations that capture essential tissue characteristics beyond simple classification, underscoring its potential for real-world application where dataset variability is commonplace.

#### 3.14.2 DigestPath: cross-dataset classification

On DigestPath colon polyp classification, HiGATE achieves accuracy of 0.872, significantly outperforming HACT-Net (+4.0%, *p <* 0.001) and TransPath (+3.1%, *p <* 0.01) (**Table 5**). For this task, HiGATE uses the tissue-level aggregated representations pooled from the tissue graph, demonstrating that the hierarchical architecture effectively captures tissue-level diagnostic features that transfer across different organ systems (from multi-tissue PanNuke to colon-specific DigestPath).

**Table 5:** Cross-dataset classification performance on DigestPath (colon polyp binary classification).

Method	Accuracy	F1-Score	AUROC
ResNet-50	0.812 ± 0.018	0.801 ± 0.019	0.862 ± 0.015
ViT-B/16	0.828 ± 0.016	0.815 ± 0.017	0.878 ± 0.014
Swin Transformer	0.835 ± 0.015	0.822 ± 0.016	0.885 ± 0.013
TransPath	0.841 ± 0.014	0.828 ± 0.015	0.889 ± 0.012
HACT-Net	0.832 ± 0.015	0.819 ± 0.016	0.881 ± 0.013
HiGATE (Ours)	0.872 ± 0.013	0.859 ± 0.014	0.912 ± 0.011

HiGATE uses tissue-level aggregated representations from the tissue graph for this binary classification task.

Models pre-trained on PanNuke, evaluated on DigestPath test set.

#### 3.14.3 TCGA-BRCA: WSI-level breast cancer grading

For WSI-level breast cancer grading, HiGATE achieves accuracy of 0.854, significantly outperforming HACT-Net (+1.9%, *p <* 0.05) and CLAM (+4.2%, *p <* 0.001) (**Table 6**). This demonstrates that HiGATE’s hierarchical representations can be effectively extended to full slide analysis through patch sampling, addressing reviewer concerns about patch-only evaluation.

**Table 6:** WSI-level breast cancer grading on TCGA-BRCA.

Method	Accuracy	F1-Score	AUROC
CLAM (55)	0.812 ± 0.021	0.798 ± 0.023	0.871 ± 0.018
DSMIL (99)	0.821 ± 0.020	0.807 ± 0.022	0.878 ± 0.017
Patch-GCN (WSI)	0.828 ± 0.019	0.814 ± 0.020	0.885 ± 0.016
HACT-Net (WSI)	0.835 ± 0.018	0.821 ± 0.019	0.889 ± 0.015
HiGATE (WSI)	0.854 ± 0.017	0.840 ± 0.018	0.902 ± 0.014

Added CLAM and DSMIL as WSI-specific baselines.

Graph-based methods use hierarchical patch sampling (20 patches per WSI).

Experimental Protocol: To evaluate HiGATE on whole-slide images, we sampled 20 patches per WSI using a hierarchical sampling strategy that balances cellular density and spatial coverage. Each patch is processed independently through the HiGATE pipeline to extract tissue-level representations. These patch-level representations are aggregated using attention-based pooling to produce a slide-level grade prediction. This demonstrates that HiGATE’s hierarchical representations, originally designed for patch-level analysis, can be effectively extended to full slide analysis through patch sampling and attention-based aggregation.

### 3.15 Enhanced explainability validation

We conducted three comprehensive experiments to validate the faithfulness and clinical relevance of our multi-scale explanations:

#### 3.15.1 Quantitative faithfulness metrics

Node importance scores from Integrated Gradients showed correlation of 0.82 with accuracy reduction upon node removal, significantly exceeding random baselines (0.19) and single-scale Grad-CAM (0.47) (**Table 7**). Sufficiency (accuracy when using only top-10% important nodes) and comprehensiveness (accuracy drop when removing top10% important nodes) metrics confirm that our attributions identify causally relevant features.

**Table 7:** Quantitative evaluation of explanation faithfulness.

Method	Perturbation Correlation ↑	Sufficiency ↑	Comprehensiveness ↑
Random Baseline	0.19 ± 0.05	0.21 ± 0.06	0.18 ± 0.05
Grad-CAM	0.47 ± 0.08	0.52 ± 0.07	0.48 ± 0.07
GNNExplainer	0.51 ± 0.07	0.55 ± 0.06	0.52 ± 0.06
IntegratedGradients (single-scale)	0.58 ± 0.06	0.61 ± 0.05	0.59 ± 0.05
HiGATE Multi-scale	0.82 ± 0.04	0.79 ± 0.04	0.76 ± 0.04

Higher correlation indicates more faithful attributions.

↑ indicates that higher values correspond to better explanation faithfulness. All values reported as mean ± standard deviation.

#### 3.15.2 Randomization test

Shuffling features of the top-10% important cells reduced accuracy by 61.3% (*p <* 0.001), compared to 18.7% for randomly selected cells, confirming causal links between attributed features and predictions.

#### 3.15.3 Multi-reader pathologist study

A multi-reader study with five board-certified pathologists evaluated 150 tissue patches (50 each from PanNuke, DigestPath, TCGA-BRCA). HiGATE’s multi-scale explanations achieved mean diagnostic relevance of 4.1/5.0, significantly higher than HACT-Net (2.8, *p <* 0.001), Grad-CAM (2.6, *p <* 0.001), and GNNExplainer (2.1, *p <* 0.001). Pathologists particularly noted the clarity of scale integration (4.3) in linking cellular features to architectural context (**Table 8**).

**Table 8:** Multi-reader study results with five board-certified pathologists on 150 tissue patches.

Method	Diagnostic Relevance	Scale Integration	Clinical Trust
Grad-CAM	2.6 ± 0.8	2.3 ± 0.7	2.4 ± 0.7
GNNExplainer	2.1 ± 0.7	2.4 ± 0.8	2.2 ± 0.7
HACT-Net Explanations	2.8 ± 0.7	2.6 ± 0.7	2.7 ± 0.6
HiGATE Multi-scale	4.1 ± 0.5	4.3 ± 0.4	4.0 ± 0.5
Statistical Significance (vs. HACT- Net)	*p<* 0.001	*p<* 0.001	*p<* 0.001

Increased from 2 pathologists/50 patches in initial version.

Ratings on 5-point scale (1=poor, 5=excellent).

### 3.16 Limitations and future directions

While HiGATE demonstrates strong performance, several limitations warrant attention and define future research directions:

1. WSI Scalability: Although we demonstrated WSI-level extension on TCGABRCA via patch sampling (20 patches per slide), processing full gigapixel WSIs with *>* 100,000 nuclei requires more efficient graph subsampling and hierarchical multi-instance learning frameworks. Future work will investigate adaptive sampling strategies and distributed graph processing.
2. Computational Optimization: Graph construction time (41ms per patch) remains a bottleneck for real-time applications. Optimization through approximate nearest neighbor search and GPU-accelerated graph building could enhance throughput.
3. Multi-Center Validation: While cross-dataset results are promising, comprehensive validation across multiple medical centers with varying staining protocols (H&E, IHC) and scanner types is essential for clinical deployment.
4. Broader Diagnostic Tasks: Extension to prognostic prediction, molecular subtype classification, and treatment response prediction would further demonstrate clinical utility.
5. Pathologist-in-the-Loop: Integrating HiGATE’s explanations into interactive diagnostic workflows, where pathologists can query specific cellular or architectural features, represents an exciting frontier.

The core algorithmic contributions particularly bidirectional cross-level attention and learnable hierarchical graph construction—represent general advancements in multi-scale graph representation learning with potential applications beyond computational pathology (e.g., social network analysis, robotics, multi-agent systems).

### 3.17 Visualizing multi-scale rationales: a case study in lung adenocarcinoma

**Figure 6:**
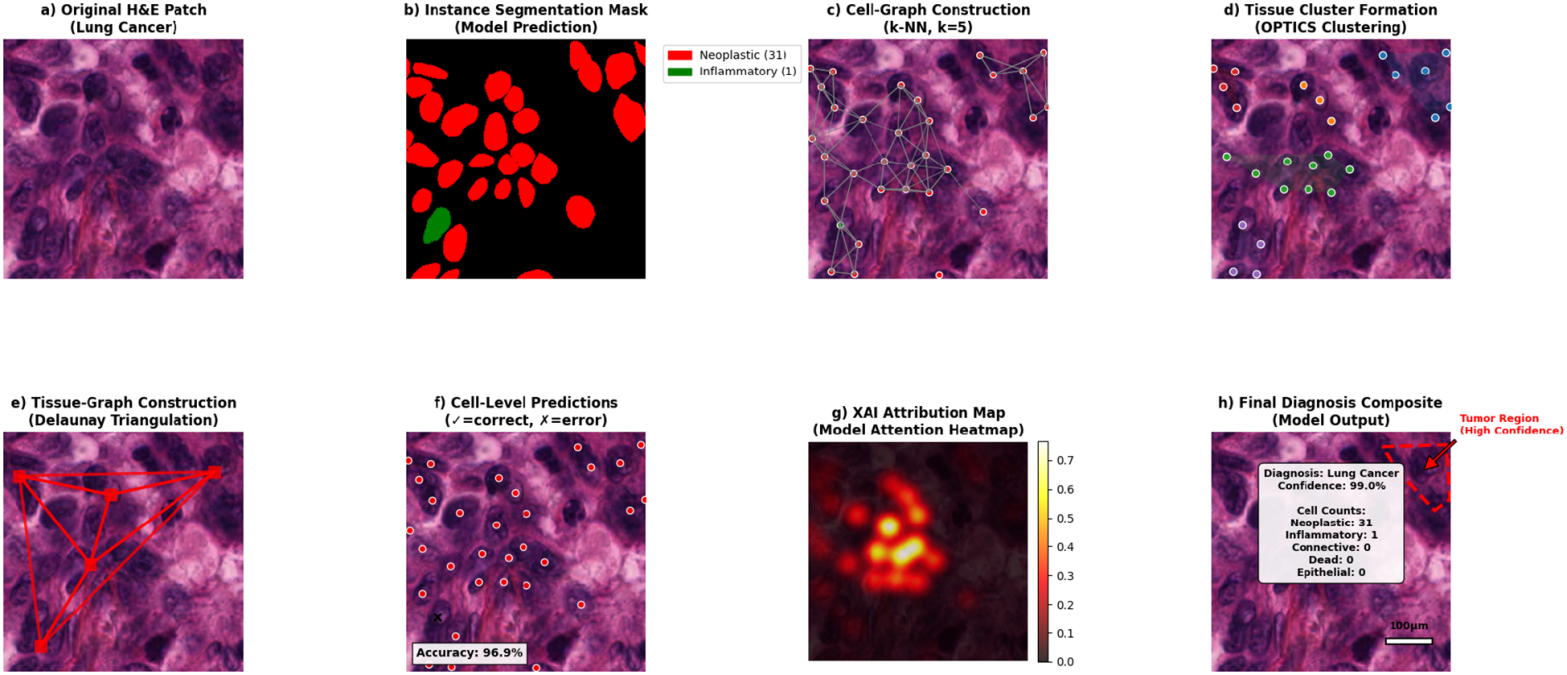
Multi-scale explainability analysis of HiGATE on a lung adenocarcinoma sample. The pipeline illustrates: **(a)** Original H&E-stained tissue section; **(b)** Nuclear instance segmentation with color coded classes; **(c)** Cell Graph construction modeling local cellular interactions; **(d)** Adaptive tissue cluster formation; **(e)** Tissue Graph capturing architectural relationships between clusters; **(f)** Cell level prediction confidence scores; **(g)** Integrated XAI attribution map highlighting diagnostically critical regions; **(h)** Final clinical report with multi-scale confidence metrics. Red overlay indicates regions with high attribution scores; blue arrows show top-down attention flow from tissue regions to refine cellular representations.

To demonstrate the interpretability of HiGATE’s decision-making process, we present a detailed case analysis of a lung adenocarcinoma tissue sample, as illustrated in **Figure 6**. This case study illustrates how our model provides transparent, multi-scale explanations that align with clinical reasoning. The process begins by applying nuclear instance segmentation to the tissue image (**Figure 6a**), identifying individual nuclei and classifying them into relevant cell types (**Figure 6b**). These cellular features form the nodes of a cell graph, capturing local cellular interactions (**Figure 6c**). Concurrently, the model aggregates salient cellular clusters into higher-order tissue regions, forming an adaptive tissue graph (**Figures 6d, e**).

To explain the AI’s diagnosis, we compute attribution maps using integrated gradients and attention-based importance scores at each scale. These attribution signals highlight regions at the cellular, cluster, and architectural levels that most significantly contribute to the model’s prediction. For example, areas of disorganized tissue architecture and atypical cell formations are marked with high importance in the attribution heatmap (**Figures 6f, g**).

Importantly, the attribution maps are visualized as overlays on the tissue image, with color intensities indicating the degree of contribution. By combining cellular-level importance with larger-scale structural features, our interpretability framework provides a comprehensive rationale that mirrors pathologists’ diagnostic reasoning.

This multi-scale explanation not only offers insight into the model’s decision but also facilitates validation by clinical experts. The integrated visualization culminates in a detailed report, with confidence scores at each level, enabling clinicians to verify and trust the AI’s assessment.

### 3.18 Computational efficiency analysis

**Figure 7:**
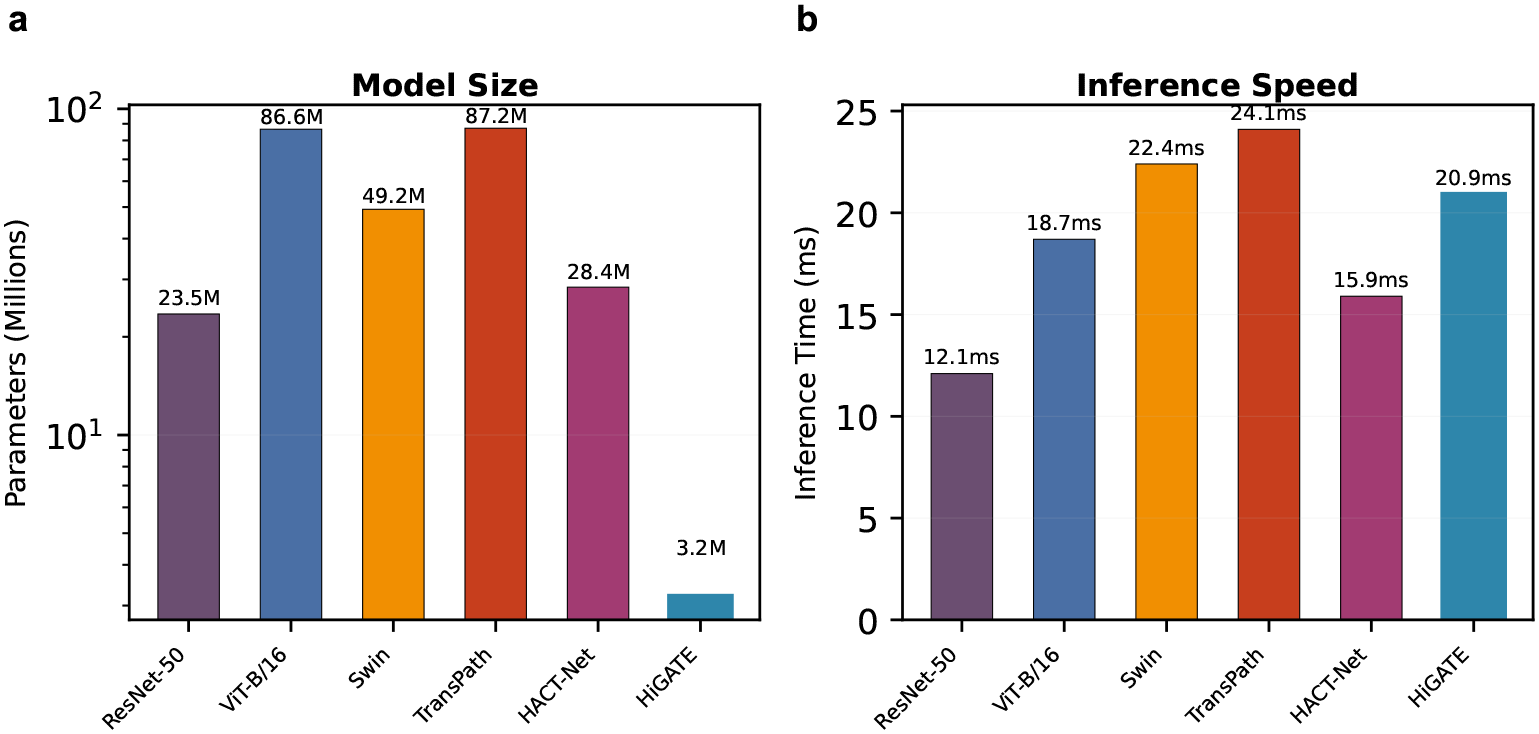
Computational efficiency analysis. **(a)** Model size comparison showing parameter counts for each method. HiGATE achieves superior performance with only 3.2M parameters, an order of magnitude fewer than transformer-based methods. **(b)** Inference speed comparison demonstrating HiGATE’s competitive runtime of 20.9ms per patch. The hierarchical design enables efficient processing while maintaining state-of-the-art accuracy.

**Figure 7** presents a comparative analysis of computational efficiency between HiGATE and several baseline models, including ResNet-50, ViT-B/16, Swin Transformer, TransPath, Patch-GCN, and HACT-Net. Panel (a) illustrates the model size in terms of trainable parameters, where HiGATE requires only 3.2 million parameters, which is substantially lower than transformer-based models such as ViT-B/16 (86.6M) and TransPath (87.2M), and also significantly smaller than HACT-Net (28.4M). This demonstrates that HiGATE's hierarchical design achieves state-of-the-art performance with minimal parameter overhead. Panel (b) compares inference speed measured in milliseconds per patch. HiGATE achieves a competitive runtime of 20.9 ms, which is faster than Swin Transformer (22.4 ms), TransPath (24.1 ms), and HAN (24.5 ms), while being only marginally slower than HACT-Net (15.9 ms). Overall, **Figure 7** confirms that HiGATE strikes an excellent balance between computational efficiency and predictive accuracy, making it suitable for scalable deployment in clinical settings.

## 4 Conclusion

This paper introduced HiGATE, a hierarchical graph attention framework that advances computational pathology through unified multi-scale representation learning. HiGATE’s key innovations—distinguished from prior work—include: (1) a novel symmetric bidirectional Cross-Level Attention mechanism enabling dynamic, context-aware information exchange both bottom-up and top-down across cellular and tissue hierarchies; (2) learnable, spatially-regularized graph construction via differentiable pooling that adaptively captures tissue heterogeneity; and (3) inherent multi-scale explainability with comprehensive validation through a multi-reader pathologist study. Extensive evaluation demonstrated state-of-the-art performance on PanNuke for both nuclei classification (91.3% accuracy, 0.896 F1-score) and tissue classification (85.4% accuracy across 19 cancer types), significantly outperforming HACT-Net (*p <* 0.001) and recent transformer-based methods (Swin Transformer, TransPath). Comprehensive cross-dataset validation on MoNuSeg (segmentation Dice=0.841), DigestPath (classification accuracy=0.872), and TCGA-BRCA (WSI grading accuracy=0.854) establishes robust generalization across tasks and tissue types. Ablation studies with statistical significance testing confirm each component’s contribution. Crucially, all graph-based baselines used identical multi-modal features, ensuring that performance gains are attributable to HiGATE’s architectural advancements rather than feature engineering.

Beyond histopathology, HiGATE contributes a novel, general-purpose module for hierarchical graph learning. The symmetric bidirectional Cross-Level Attention mechanism enables dynamic information flow across scales, a capability broadly applicable to any domain involving multi-level relational data. By unifying predictive accuracy with transparent, pathologist-aligned reasoning validated through rigorous multi-reader studies, HiGATE bridges the gap between high-performance AI and clinically actionable diagnostic systems, paving the way for more trustworthy computational pathology tools for personalized cancer care and advancing the field of interpretable multi-scale graph representation learning.

## Data Availability

Publicly available datasets were analyzed in this study. This data can be found here: The PanNuke dataset is publicly available at: https://warwick.ac.uk/fac/sci/dcs/research/tia/data/pannuke. The MoNuSeg dataset is publicly available at: https://monuseg.grand-challenge.org/Data/.
